# Evaluation of Physicochemical and Microbial Properties of Extracts from Wine Lees Waste of Matelica’s Verdicchio and Their Applications in Novel Cosmetic Products

**DOI:** 10.3390/antiox12040816

**Published:** 2023-03-27

**Authors:** Lucrezia Di Nicolantonio, Marta Ferrati, Maria Cristino, Dolores Vargas Peregrina, Marco Zannotti, Luca Agostino Vitali, Sonia Ilaria Ciancia, Rita Giovannetti, Stefano Ferraro, Susi Zara, Valentina Di Valerio, Amelia Cataldi, Maria Rosa Gigliobianco, Roberta Censi, Piera Di Martino

**Affiliations:** 1 Cosmetology Laboratory, University of Camerino, 62032 Camerino, Italy; 2 Recusol Srl, 62032 Camerino, Italy; 3 Chemistry Interdisciplinary Project (ChIP), School of Pharmacy, University of Camerino, Via Madonna delle Carceri 9/B, 62032 Camerino, Italy; 4 Chemistry Interdisciplinary Project (ChIP), School of Science and Technology, Chemistry Division, University of Camerino, Via Madonna delle Carceri, 62032 Camerino, Italy; 5 Microbiology Unit, School of Pharmacy, University of Camerino, via Gentile III da Varano, 62032 Camerino, Italy; 6 Department of Pharmacy, University “G. d’Annunzio” Chieti-Pescara, Via dei Vestini 31, 66100 Chieti, Italy; 7 Department of Medicine and Aging Sciences, “G. d’ Annunzio” Chieti-Pescara, Via dei Vestini 31, 66100 Chieti, Italy

**Keywords:** wine lees, ultrasound assisted extraction, yeast extract, vinification lees, by-product valorization, extraction methods, wine waste, cosmetic formulations

## Abstract

Wine lees are sediments deposited on the walls and bottom of barrels resulting from wine fermentation and mainly consist of yeasts. *Saccharomyces cerevisiae* extracts, rich in beneficial components for the skin, have already been used in cosmesis, while wine lees have not been well exploited by the cosmetics industry yet. The aim of this work was the full characterization of the wine lees from Verdicchio’s wine, with the aim to exploit it as a beneficial ingredient in new cosmetic products. After mapping the microbial composition of the sample waste, the parameters for the sonication extraction process were optimized and the physicochemical properties of the extract were analyzed. The efficiency of the aqueous extraction—and in particular the yeast cell lysis necessary for the release of proteins from the cell—was assessed by evaluating cell shape and size, and protein release, under scanning electron microscopy (SEM), dynamic light scattering (DLS) and Bradford’s protein assays. Thus, the total phenol content and antioxidant capacity of the supernatant recovered from native and sonicated lees were determined by Folin–Ciocalteu’s and spectrophotometric assays, respectively. To quantify the heavy metals and highlight the presence of microelements beneficial for the skin, inductively coupled plasma-mass spectrometry (ICP-MS) was applied. In vitro metabolic activity and cytotoxicity were tested on both HaCat keratinocytes and human gingival fibroblasts, showing that wine lees are safe for skin’s cells. The results show that sonicated lees appear to be more interesting than native ones as a consequence of the release of the active ingredients from the cells. Due to the high antioxidant capacity, content of beneficial elements for skin and an appropriate microbiologic profile, wine lees were included in five new solid cosmetic products and tested for challenge test, compatibility with human skin, sensory analysis, trans epidermal water loss (TEWL) and sebometry.

## 1. Introduction

During the winemaking process, thousands of tons of winery waste is generated. Among the various sub-products of the wine industry—such as grape pomace, stalks and dewatered sludge—wine lees represents around 12% of the total waste, and it is defined as the residue formed at the bottom of recipients containing wine, after fermentation, during storage or after authorized treatments, as well as the residue obtained following filtration or centrifugation of this product. Wine lees are among the most underexploited residues in the enological industry [1,2,3].

There are two types of classifications for lees; on the one hand, they are classified depending on the stage of vinification: first and second-fermentation lees, which are formed during the alcoholic and malolactic fermentations, respectively, and aged wine lees formed during wine aging in wood barrels. On the other hand, they can also be classified depending on particle size: heavy lees (between 100 µm and 2 mm) and light lees (<100 µm).

Wine lees can be found as semi-solid residue constituted by liquid and solid lees. The solid fraction is a combination of yeasts, organic acids (mainly tartaric acid), insoluble carbohydrates (such as cellulosic or hemi-cellulosic materials), inorganic salts, lignin, proteins, phenolic compounds, pulp and other parts of the grape. The liquid fraction is mainly composed of ethanol and organic acids, such as lactic and acetic acids.

Quantitative and qualitative characteristics of winery wastes vary widely according to the type of wine produced, the winemaking technologies employed, the size of winemaking facilities and the season [4,5].

Yeasts are the main component of the lees, in fact they are the main responsible for the process of alcoholic fermentation in the production of wine, together with many microorganisms including other fungi and bacteria.

Yeasts have already been exploited by the cosmetics industry: in particular, some studies report that they have positive effects on the skin, demonstrated by evaluating transepidermal water loss (TEWL), skin moisture (SM) and skin microrelief (SMR) [6].

*Saccharomyces cerevisiae* extract in particular has a prominent role among biotechnological raw materials in cosmetic products, because it is rich in amino acids and peptides that can have moisturizing properties, as well as proteins and polysaccharides, which are beneficial for cell renewal effects. These extracts are also rich in components that provide benefits such as the prevention of photoaging and oxidative stress diseases; there are also molecules such as vitamins B6 and B12; minerals (enzyme co-factors) such as zinc, copper and manganese; phytosterols and phenolics, including catechins and trans-resveratrol with antioxidant activity, as they are produced by *Saccharomyces cerevisiae* in the adaptive response to oxidative stress. As such, these components can improve photoaged skin [7].

By-products derived from wine-making, like lees, pomace and stalks, are subject to a defined method of management, which includes the obligation of total or partial delivery to the distillery or disposal under control for alternative uses [8]. Usually, wineries send wine lees to distilleries, which then use the material to recover alcohol; part of this waste is used by the winery itself to separate spent yeast and reuse it in the subsequent wine production process, as these fungi act as fermentation activators. Alternatively, wine lees are sent to companies that use them for the recovery of organic acids as tartaric acid.

The increasing attention to the circular economy represents an opportunity to both reduce disposal costs and improve the environmental and economical sustainability of the wine supply chain. In this scenario, the exploitation of grape pomace products such as spirits and grape seed oil—and for extracting additives like polyphenols—is already well established [9,10,11], while wine lees, the second largest by-product of winemaking, have so far received only a modest attention regarding their possible valorization.

The treatments obtained in the lees mainly concern the recovery and transformation of compounds with high added value. Wine lees are traditionally fundamental raw materials for the production of ethanol and tartaric acid [12], and they are used for the production of methane-rich biogas through anaerobic digestion [13]. Proteins, lipids and polysaccharides are found mainly inside yeast cells and on the cell wall. At the end of the alcoholic fermentation process, yeasts die and a process of cell lysis begins. This process allows the release of cytoplasmic material (peptides, fatty acids, nucleotides, amino acids) and cell wall compounds (mannoproteins) [14,15]. Autolysis is a very long and slow process, but in industrial processes it can be induced by physical inductors (rise in temperature, alternate freezing and thawing and osmotic pressure), chemical inductors (pH, detergents and antibiotics) or biological inductors (aeration and starvation) [16].

Some studies report the extraction of bioactive molecules of cosmetic interest, such as polyphenols (flavonols and phenolic acids), squalene or for the recovery of mannoproteins and beta-glucans [5,17,18,19,20,21]. Usually, the recovery of these substances is favored by pre-treatments that allow their release.

Even though the beneficial properties of lees for the skin have already been described [22,23,24], there are few cosmetic industries that have used this agro-industrial waste in cosmetic formulations, and in many cases wine lees have been used as is, without transformation.

The aim of this study is the optimization of extraction and the full characterization of a sonicated extract obtained from Matelica’s Verdicchio wine lees. A preliminary microbiological analysis is intended to exclude harmful effects of wine lees and highlight the benefit of obtaining raw material by yeast cells extract, favoring the breakdown of cells with ultrasound. This works is also intended to verify if there are differences between the native and sonicated lees, and to understand how to better exploit the beneficial properties of wine lees.

Finally, an attempt is made to formulate a set of cosmetic products containing this waste, with a low environmental impact. Final original solid cosmetic formulations are thus proposed, with the intention of verifying positive cosmetic effects on the skin, in a context where solid waste reduction and recycling are becoming increasingly important for consumers who care for the environment

## 2. Materials and Methods

### 2.1. Wine Lees

**Figure 1:**
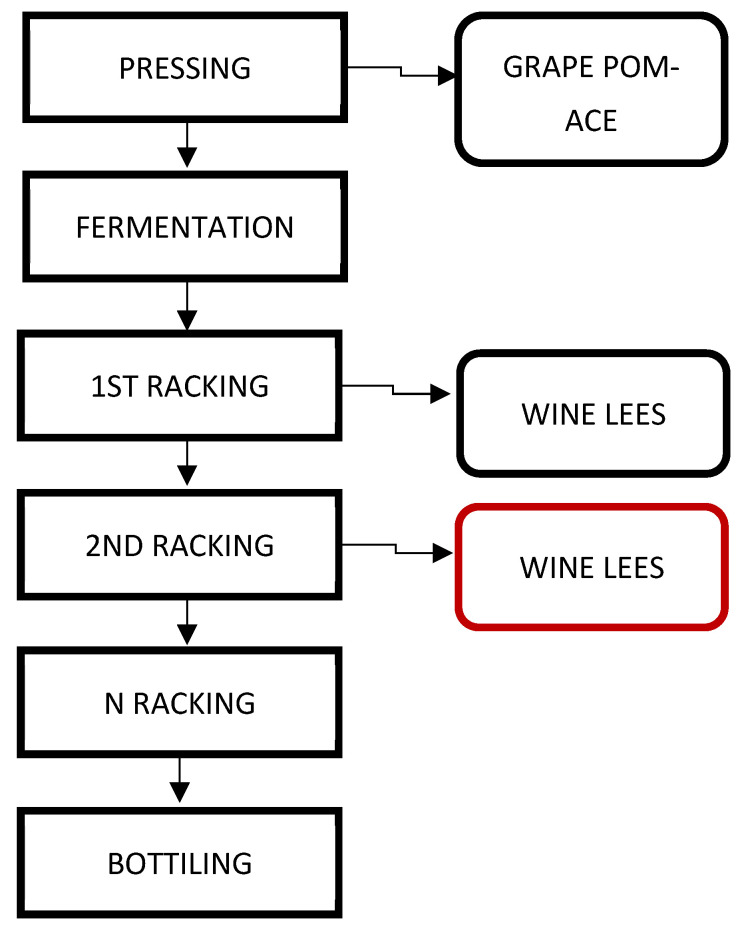
Vinification process from which wastes are produced, Inside the red box, the type of less received from Belisario Winery.

The native wine lees (NWLs) used in this study were kindly supplied by Belisario’s winery (Matelica, Italy) and are derived from the vinification process of 2020 Matelica’s Verdicchio. This wine is produced starting from the first phase of the harvesting of Verdicchio’s grapes, which are then pressed. Briefly, grape pomaces obtained by pressing are removed from the must, which, at this point, undergoes the process of alcoholic fermentation. After the fermentation process, the wine is poured in other casks to separate the components which gradually decant. Lees obtained from the first racking are usually richer in yeasts, whereas the ones obtained from the second racking have a lower concentration of yeasts, in detriment of the higher quantity of tartaric acid, which crystallizes due to the acid pH after salification with potassium ion. NWLs provided for this study comes from the second racking of the wine (Figure 1) to highlight the potential of exploiting a waste considered of lower quality.

### 2.2. Reagents and Materials Other Than Wine Lees

Deionized water was produced with a G3 RO CUBIC-S2 demineralizer (Gamma3, Castelverde, Italy). Hexane, acetonitrile and ethanol were purchased from Carlo Erba Reagents (Cornaredo, Mi, Italy). Reagents used for the antioxidant assays, such as 2,2- diphenyl-1-picrylhydrazyl (DPPH), 6-hydroxy-2,5,7,8-tetramethylchroman-2-carboxylic acid (Trolox), 2,2&39;-azino-bis(3-ethylbenzothiazolin-6-sulfonic acid) (ABTS), manga-nese dioxide, 2,4,6-Tri(2-pyridyl)-s-triazine (TPTZ), HCl 37%, sodium acetate, iron tri-chloride, Folin reagent, sodium carbonate and gallic acid were purchased from Sigma-Aldrich (Stenheim, Germany). A soil DNA isolation plus kit was purchased from the Norgen Biotek Corporation (Thorold, ON, Canada). The human keratinocyte cell line (HaCaT) was purchased from AddexBio, catalog numberT0020001, (AddexBio, San Diego, CA, USA). Reagents for metabolic activity assays, such as dimethyl sulfoxide (DMSO) and 3-(4,5-dimethylthiazol-2-yl)-2,5-diphenyltetrazolium bromide (MTT), were purchased from Merck Life Science (Milan, Italy); medium and sera for cell cultures, such as Dulbecco’s modified Eagle’s medium (DMEM), foetal bovine serum (FBS) and penicil-lin/streptomycin were purchased from Euroclone (Milan, Italy); tissue culture-treated plates were purchased from Falcon^®^, Corning Incorporated, New York, NY, USA. CytoTox 96^®^ non- radioactive assay (Promega Corporation, Fitchburg, WI, USA) was used to determine Lactate dehydrogenase (LDH) release (cytotoxicity assay). Ex Taq^®^ DNA Polymerase, dNTPs (10 mM) and 10X Buffer (Mg 2 mM) were purchased from Takara Bio Inc. (San Jose, CA, USA). Primer F (pro 341 F) and Primer R (pro 805 R) were from Sigma-Aldrich (Stenheim, Germany). *Klebsiella pneumonie* preparation, strain ATCC13883, was made in a laboratory in 2019. De Man, Rogosa and Sharpe agar (MRS), Tryptic Soy Agar (TSA) and Sabouraud Dextrose Agar (SDA), MacConkey Agar (MCA), Pseudomonas Cetrimide Agar (PCA) and Mannitol Salt Agar (MSA) media were from Thermo Fisher Scientific (Waltham, MA, USA). Chloramphenicol solution (20 mg/mL) was pre-pared using chloramphenicol from Sigma-Aldrich (Stenheim, Germany) and 95% etha-nol. GenElute bacterial genomic DNA Kit was purchased from Sigma-Aldrich (Stenheim, Germany). Squalene analytical standard was purchased from Merck KGaA, (Darmstadt, Germany).

### 2.3. Microbiological Analysis of Native Wine Lees

#### 2.3.1. Extraction of Total DNA

To determinate the bacterial and fungal species total content in NWLs, the total DNA was extracted using a Soil DNA isolation plus kit (Nalgene, Waltham, MA, USA), following the manufacturer’s instructions and protocol. Starting material volume of NWLs was 400 μL. The final volume of the eluate from the column 50 μL. Four replica samples were prepared, following the same procedure described above, and they were stored at −20°C.

#### 2.3.2. Quality Check by PCR and Agarose Gel Electrophoresis

A PCR protocol was developed to make the quality check by amplification of a portion of the 16S rRNA gene. The protocol developed is standard and very much like the one suggested by the biotech company committed in the NGS workflow analysis (BMR Genomics, Padova, IT). A Bio-rad iCycler PCR system was used. A *Klebsiella pneumoniae* total chromosomal DNA preparation already available in the lab was used as the positive control.

Reaction mixtures for amplification contained 1X TaKaRa Buffer (TaKaRa Bio, Kusatsu, Japan), 2 mM MgSO_4_, deoxynucleotides—0.2 mM each, 0.5 Units Ex Taq Polymerase (TaKaRa Bio, Kusatsu, Japan) and 5 µL of the genomic DNA prep. Oligonucleotide primers (Pro341F: 5′-CCTACGGGNBGCASCAG-3′ and Pro805R: 5′-GACTACNVGGGTATCTAATCC-3′) were added at a final concentration of 0.4 µM each [25]. The final reaction volume was 25 µL. The negative control containing no DNA was subjected to the same procedure to exclude or detect any possible contamination. Thermal cycling condition were as follows: 1 cycle at 94 °C for 1 min, then 25 cycles (94 °C for 30 s, 55 °C for 30 s; 68 °C for 45 s); 1 cycle at 68 °C for 7 min.

After amplification, 5 μL of loading dyes were added to each PCR tube. Aliquots of each sample of 20 μL and 6 μL of 1 kb standard were loaded on a 1.2% agarose gel, prepared in 0.5X buffer of TBE, embedding ethidium bromide (0.5 µg/mL) into the gel matrix during gel casting. Amplicons were visualized on a TFX-20.M UV transilluminator.

#### 2.3.3. Preparation and Shipment of Samples

Aliquots of 45 μL of sample obtained in “2.3.1” were transferred into 4 PCR tubes and were sent to the biotech company BMR Genomics (Padua, Italy) for analysis; two duplicates (named GDA and GDB) were sent for 16S rDNA-based NGS, and the other two (named GDC and GDD) for 18S rDNA ITS NGS sequencing.

#### 2.3.4. Preparation and Inoculation of Microbial Growth Media

To verify the presence of living microbial cells in NWLs that may pose a potential health concern, a 1:10 (F1:10) and a 1:100 (F1:100) dilution of lees in saline were prepared for inoculum on different enrichment solid media (Tryptic Soy Agar—TSA; De Man, Rogosa and Sharpe agar—MRS and Sabouraud Dextrose Agar—SDA). Aliquots of 100 μL of F1:10 solutions were inoculated onto 2 TSA plates, 2 MRS plates, 1 SDA plates and 1 SDA plate with chloramphenicol (20 mg/L final concentration). The latter was added to inhibit bacterial growth. A total of 100 μL aliquots of F1:100 were inoculated following the same scheme. All plates were checked after 7 days under the incubation conditions summarized in Table 1.

#### 2.3.5. Bacterial Population Enrichment

Single colonies from the enrichment media plates (2.3.4) were transferred to 4 TSA plates, each divided into 16 quadrants, to grow each single isolate. Plates were incubated for 24 h at 37 °C.

#### 2.3.6. Microbial Growth on Selective and Differential Media

The bacterial colonies grown in the above sectorial plates (2.3.5) were replica plated on-to selective and differential media MacConkey Agar (MCA), Plate Count Agar (PCA) and Mannitol Salt Agar (MSA), for the isolation and identification of specific microorganisms [26], respectively of *Enterobacteriaceae* (e.g., *E. coli*), *Pseudomonas aeruginosa* and *Staphylococcus aureus*, [27], the same addressed for in the microbiological quality of the cosmetic products (as per ISO 17516:2014) [27]. Specifically, 4 plates were prepared for each type of media, and microbial growth was evaluated after 48-h incubation at 37 °C.

#### 2.3.7. DNA Extraction by GenElute Bacterial Genomic DNA Kit

For the preparation of the chromosomal bacterial DNA from isolates (2.3.5), GenElute bacterial genomic DNA Kit (Merck KGaA, Darmstadt, Germany) was used following the protocol for gram-positive bacteria, which is more efficient in cell disruption and DNA release. Single colonies of each isolate grown in all TSA plates (2.3.5) were pooled together into 500 μLl of TSB, obtaining a polymicrobial culture. After 1.5 h of incubation at 37 °C, total DNA was extracted. At the last step of the procedure of purification the final elution volume was 200 μL.

#### 2.3.8. Quality Check by PCR and Agarose Gel Electrophoresis

Extracted DNAs (2.3.7) were subjected to 16S ribosomal RNA gene region PCR amplification using the same protocol applied for quality check (2.3.2. and 4). Additionally, a genomic DNA prep from a reference strain of *Klebsiella pneumoniae* was used as positive control. A reaction with no DNA was added as a negative control. After amplification, 7–8 μL from each sample were loaded on the agarose gel. After electrophoresis (6–7 Volts/cm), the banding patterns were visualized by UV irradiation.

#### 2.3.9. Preparation and Shipment of Samples for NGS Analysis

A total of 30 μL of Genomic DNA 1 and Genomic DNA 2 were transferred into PCR tubes and sent to BMR Genomics (Padua, Italy) for 16S rDNA NGS analysis.

#### 2.3.10. NGS Bioinformatic Analysis Using DADA2

Analysis of generated sequences by 16S and 18S ITS NGS at step 2.3.1 or 2.3.7 was performed using the DADA2 pipeline [28]. Taxonomic assignment of sequences was done using the SILVA reference database for 16S rDNA sequences, compiled for use in the DADA2 pipeline and downloaded from https://doi.org/10.5281/zenodo.4587955 (accessed on 13 October 2022) [29], while for 18S ITS, the training set was from https://doi.org/10.5281/zenodo.4310151 (accessed on 13 October 2022). The DADA2 pipeline was run using Galaxy with default settings [30].

### 2.4. Ultrasound-Assisted Yeast Lysis of NWLs and Physicochemical Characterization of the Aqueous Extract

#### 2.4.1. Ultrasound-Assisted Yeast Lysis of NWLs

To break up yeast cells of NWLs for the release of cytoplasmatic and wall-associated material, an ultrasonic processor (US, Sonicator Q500, QSonica, Newton, CT, USA), with a 19-mm probe made of titanium, was used. Several preliminary conditions were tested in order to optimise a fast and effective cell break, easily scalable for further industrial purposes, and finally, the following conditions were retained. NWL was diluted in 1:10 (g/g) of water as solvents. Samples were sonicated at a constant frequency of 20 kHz for 5 min. The energy input was controlled by setting the amplitude at 99%, by considering that the total energy of the instrument is 500 W. The probe was submerged in a 250-mL beaker containing 100 g of diluted lees, and the beaker was placed in an ice bath and carefully fixed during sonication.

The cell lysis was confirmed by examining small aliquots under a Dialux 22 microscope (Ernst Leitz, Wetzlar, Germany) at a magnification of 100×.

All the obtained materials were characterized. This product will be identified as sonicated wine lees (SWL).

#### 2.4.2. Scanning Electron Microscopy (SEM)

NWL and SWL were analyzed under scanning electron microscopy to determine the particle shape and size of the cells. A drop of the suspensions (from both NWL and SWL) was deposited on a stub leaving for spontaneous solvent evaporation necessary for the analysis. Samples were fixed on the stub for the FE-SEM using a double-sided carbon pad and metallized by depositing a 10 nm chromium film with a metallizer (Quorum Technologies’ Q150T, Laughton, UK). For the SEM analysis, a ZEISS microscope (FE-SEM SIGMA 300) was used (Oberkochen, Germany).

#### 2.4.3. Dynamic Light Scattering (DLS)

The particle size and the PDI of NWL and SWL were characterized by dynamic light scattering (DLS) (Zetasizer Nano-S90, Malvern instruments, Malvern Panalytical, Malvern, UK) at a fixed 90° scattering angle at 25 °C. The samples were suspended in ultrapure water and measurements were performed in triplicate.

#### 2.4.4. Bio-Rad Protein Assay

The determination of the total soluble proteins content in NWL and SWL was carried out by Bradford’s method using the BioRad Protein Assay reagent [31]. Bovine serum albumin (BSA) was used as standard. To prepare the BSA standard solution, 2.8 mg of BSA were weighed and dissolved in 1 mL of deionized water. To obtain a concentration of 0.1 mg/mL, 20 μL of solution were diluted with 540 μL of water. The calibration curve was obtained using 6 dilutions of BSA. The equation relative to the calibration curve obtained from the absorbance read by the spectrophotometer and related to the BSA standard solution concentration is y = 0.0481x + 0.075 (R^2^ = 0.998). The absorbances (595 nm) were measured at 15 min and one hour after sample preparation. For the measurements, a SHIMADZU UV-1700 PharmaSpec spectrophotometer (Kyoto, Japan) was used. This analysis was performed on the sample supernatants after centrifugation at room temperature at 4000 rpm for 15 min in a Heraeus Megafuge 1.0R (Kendro Laboratory Products GmbH, Hamburg, Germany). The lyophilized supernatants were suspended in water, and the difference between the supernatant of the NWL and SWL was evaluated.

#### 2.4.5. Quantitative Element Analysis

The samples (NWL and SWL) were prepared by homogenizing the lees with the T 25 digital ULTRA-TURRAX homogenizer (Ika, Leiden, The Netherlands) and then centrifuged at room temperature for 15 min at 4000 rpm. The lyophilized supernatants of NWL and SWL were first dispersed in Grade 1 water (Millipore Milli-Q Advantage A10 system, resistivity 18.2 MΩ cm) and the optimized digestion of the samples was carried out by placing the sample in a Teflon digestion vessel, followed by 1 mL of HNO**_3_** (65%) and 4 mL of H**_2_**O**_2_** (20%) Suprapure grade. A total of 50 μL of Be, Ru and Au solution (2 mg/L) was added as the recovery standard. The vessel was immediately sealed and placed in a microwave closed vessel system (Berghof Speedwave four, Berghof, Eningen, Germany) for digestion. The microwave digestor program is indicated in Table 2. Digested solutions were transferred to a 10 mL volumetric flask and diluted with ultrapure water, then diluted again 10 times to obtain a solution with the correct acid concentration.

The concentrations of elements in the processed samples from the first and the second preparation were measured by an Agilent ICP-MS (7500cx series) with the following operating conditions: power 1550 W, carrier gas 1.03 L/min, make-up gas 0.00 L/min, sample depth 7.5 mm, nebulizer pump 0.1 r.p.s. and spray chamber temperature 2 °C. The 7500cx series instrumentation can be operated in NoGas/He mode, in order to overcome most of the polyatomic interference by the collision cell. A typical performance test in He mode was performed with a 1 ppb tune solution and gave the following results: He flux 3.0 mL/min, *m*/*z* 9 (1000 cps), *m*/*z* 45 (1600 cps), *m*/*z* 115 (1500 cps), *m*/*z* 140 (2000 cps) and *m*/*z* 209 (1200 cps). A solution containing Sc, In, Ce and Bi (10 mg/L) was used as the internal standard for ICP-MS measurements. Calibration curves for investigated elements were obtained using aqueous (1.0% nitric acid) standard solutions prepared with appropriate dilution of stock standards (Fluka Analytical, Aldrich, Milan, Italy). The calibration straight was made with the following solutions for microelements (Li, Be, B, Al, Ti, V, Cr, Mn, Fe, Co, Cu, Zn, Ga, As, Se, Rb, Sr, Mo, Ru, Pd, Ag, Cd, Sn, Sb, Cs, Ba, Au, Tl, Pb, U): 0.01 ppb; 0.10 ppb; 1.00 ppb; 5.00 ppb; 10.0 ppb; 50.0 ppb; 100.0 ppb; 500.0 ppb. For the calibrations straight of macroelements (Na, Mg, P, S, K, Ca) the solutions used were: 0.50 ppm; 1.00 ppm; 2.50 ppm; 5.00 ppm; 10.0 ppm; 25.0 ppm; 50.0 ppm. Solutions for the calibration straight of Hg element was as followed: 0.1 ppb; 0.5 ppb; 1.0 ppb; 5.0 ppb; 10.0 ppb.

#### 2.4.6. Total Phenol Content (Folin-Ciocalteu Assay)

The total phenol content (TPC) of NWL and SWL was determined according to the Folin–Ciocalteu spectrophotometric method [32]. The reagent was prepared adding 6 mL of Folin’s reagent in 24 mL of distilled water. For the samples, the lyophilized supernatants of NWL and SWL were used. A total of 100 μL of each sample was added in the first row of a 96-well microplate, including the gallic acid control solution made with 1 mg of gallic acid in 1 mL of distilled water, which is used like a standard. Serial dilutions of 1:2 (*v*/*v*) were made. In the last well of the 96-plate, a control with deionized water was prepared. Subsequently, 150 μL of the reagent and 50 μL of a saturated Na**_2_**CO**_3_** solution, previously prepared, were added to each well. The plate was incubated at 37 °C for 30 min, and then it was analyzed in a microplate reader (FLUOstar Omega, BMG Labtech GmbH, Ortenberg, Germany). The absorbance of each well was determined at 765 nm, and the measurements were compared to the calibration standard solution of gallic acid (GA). The final results of the assay have been calculated using Graph Pad Prism 9 and they were expressed in mg of gallic acid equivalent/g of sample (mg GAE/g). All the measurements were performed in triplicate.

#### 2.4.7. Antioxidant Assays

The antioxidant activity was determined on the lyophilized supernatant of NWL and SWL. It was evaluated by measuring 1,1-diphenyl-2-picrylhydrazyl (DPPH•) radical scavenging activity, 2,2′-azino-bis (3-ethylbenzothiazoline-6-sulphonic acid) (ABTS•) radical scavenging capacity and Ferric reducing antioxidant capacity (FRAP). 6-hydroxy-2,5,7,8-tetramethylchroman-2-carboxylic acid (TROLOX) was used in every assay as standard for the calibration curve. The results of the assays were calculated using Graph Pad Prims 9, and the values were expressed in IC50, which represents the concentration of the samples required for a decrease of 50% of the initial DPPH, ABTS or iron concentrations, and in μmol or mg of Trolox equivalent/g of sample [32].

##### DPPH Radical Scavenging Method

DPPH (1.2 mg) was dissolved in 30 mL of ethanol, obtaining a purple solution. For the calibration standard, a solution of Trolox in ethanol (1 mg/mL) was prepared, and the diluted 1:10 (*v*/*v*) in ethanol. 100 μL of each sample were added in the first row of a 96-well microplate, including the Trolox. Serial dilutions 1:2 (*v*/*v*) were made. Deionized water was used as control. A total of 150 μL of DPPH solution, previously prepared, was added in every well, and the plate was incubated in the dark at 37 °C. After 30 min of incubation, the absorbance of each well was measured at 517 nm with the microplate reader (FLUOstar Omega, BMG Labtech GmbH, Ortenberg, Germany). All the measurements were performed in triplicate.

##### ABTS Radical Scavenging Method

ABTS (9.8 mg) and 0.6 g of MnO**_2_** were added in 3.6 mL of H**_2_**O, obtaining a green solution, which was incubated in a dark place for 20 min. After this period, the solution was filtered and 1 mL of this was added in 30 mL of water. The Trolox solution was prepared following the same method used for DPPH assay. A total of 100 μL of each sample was added in the first row of the 96-well microplate, including the Trolox solution. Serial dilutions 1:2 (*v*/*v*) were made, and in the last well of the plate a control was made with deionized water. A total of 150 μL of the ABTS solution was added in every well, and the plate was incubated in the dark for 30 min, at 37 °C. After this period, the absorbances of each well were measured at 734 nm, with the same microplate reader as the DPPH assay. All the measurements were performed in triplicate.

##### Ferric Reducing Antioxidant Power (FRAP)

An acetate buffer pH 3.6 was prepared with 2.46 g of sodium acetate added in 80 mL of distilled water. This solution was acidified with acetic acid until pH 3.6 and made up to 100 mL with water. A solution of 15.6 mg of 2,4,6-tripyridil-s-triazine (TPTZ) in 5 mL of HCl 40 mM and another solution of 16.2 mg of FeCl**_3_** in 5 mL of HCl 40 mM, were added to 50 mL of the acetate buffer to obtain the reagent FRAP. A Trolox solution for the calibration curve was prepared with the same procedure for DPPH and ABTS assays. A total of 50 μL of each sample was added in the first row of the 96-well plate microplate, including the standard Trolox. Serial dilutions 1:2 (*v*/*v*) were made, and in the last well a control was prepared with deionized water. A total of 175 μL of the FRAP solution was added to each well, and the plate was incubated for 60 min at 37 °C. After this period, the absorbances were measured at 593 nm, with the same microplate reader as the other assays. All measurements were performed in triplicate.

#### 2.4.8. Quantitation of Squalene by High-Performance Liquid Chromatography Coupled with Diode Array Detection

Squalene was obtained by a lipid extraction with a sonication process. For the extraction, 1 g of NWL was sonicated in 100 mL of *n*-hexane used like solvent, and the extraction was made in a water bath to avoid increasing the temperature. The sonication was made with a duty cycle with an active interval of 8 sec, using the 19 mm titanium probe, for 29 min, with an amplitude of 97%. After the sonication, the sample was centrifuged at 4000 rpm for 15 min and the solvent was removed under vacuum (40 °C).

The identification and quantitation of squalene were carried out using an HPLC Agilent (1200 Series chromatograph), coupled with a diode array detector (DAD). Chromatographic separation was performed using a ALLTIMA (C18, 150 mm × 4.6 mm) with a particle diameter of 5µm. The column temperature was set at 30 °C. The analysis was performed in presence of an elution gradient with a mobile phase of acetonitrile at a constant flow rate of 1.5 mL/min [33]. Squalene standard was added in 2 mL of mobile phase and then sonicated for 10 min to allow complete solubilization. Before injection, samples were filtered through a 0.45 μm membrane filter to remove undissolved particles. Five µL of squalene standard and 5 µL of each sample were injected into the HPLC. Analytes were monitored with a UV detector at a wavelength of 195 nm. Quantitative analyses in UV/Vis-based detection systems were performed using a linear calibration curve generated with the squalene standard.

### 2.5. Determination of Cell Metabolic Activity and Cytotoxicity

#### 2.5.1. Cell Culture and Treatments

HaCaT cell line was cultured in DMEM high glucose supplemented with 10% of fetal bovine serum (FBS) and 1% of penicillin/streptomycin. Human gingival fibroblasts (HGFs) were extracted as previously reported [34]**,** after having received the approval of the Local Ethical Committee of the University of Chieti (Chieti, Italy; approval number. 1173, approved on 31 March 2016). Both the cell cultures were kept at 37 °C in a humid atmosphere with CO**_2_** 5%.

HaCat and HGFs were seeded at density of 8000 and 6700, respectively, in a 96-well tissue culture-treated plate, and then allowed to adhere for 24 h. NWL and SWL were previously dispersed in sterile water (starting solution 3 mg/mL) and then administered to Ha-Cat and HGFs at 1, 5, 10 and 20% in culture medium, for 24, 48 and 72 h.

#### 2.5.2. Cell Metabolic Activity Test (MTT)

HaCaT cell line was cultured in DMEM high glucose supplemented with 10% of fetal bovine serum (FBS) and 1% of penicillin/streptomycin. Human gingival fibroblasts (HGFs) were extracted as previously reported [33], after having received the approval of the Local Ethical Committee of the University of Chieti (Chieti, Italy; approval number. 1173, approved on 31 March 2016). Both cell cultures were kept at 37 °C in a humidified atmosphere with CO**_2_** at 5%.

HaCat and HGFs were seeded at density of 8000 and 6700, respectively, in a 96-well tissue culture-treated plate and then let adhere for 24 h. NWL and SWL were previously dispersed in sterile water (starting solution 3 mg/mL) and then administered to Ha-Cat and HGFs at 1, 5, 10 and 20% in culture medium, for 24, 48 and 72 h.

#### 2.5.3. Cytotoxicity Assay

To assess cytotoxicity occurrence, the release of LDH into cell supernatants was quantified by the CytoTox 96^®^ non- radioactive assay, which uses a 30-min coupled enzymatic assay, after 24 and 48 h of treatment. The optical density was measured at 490 nm with a correction at 690 nm, by means of a spectrophotometer (Multiskan GO, Thermo Scientific, Waltham, MA, USA). The percentage of released LDH was therefore normalized on the optical density values obtained from the metabolic activity assay.

### 2.6. Statistics

Statistical analysis was performed using the GraphPad 7 software (GraphPad Software, San Diego, CA, USA) by means of ordinary one-way ANOVA, followed by post−hoc Tukey’s multiple comparisons test. Values of *p* < 0,05 were considered statistically significant.

### 2.7. Formulation of Cosmetic Products

Five cosmetic formulations containing the SWL were developed. For the formulations, the whole ingredient recovered after the sonication was used composed of both the insoluble particles. According to the International Nomenclature Cosmetic Ingredients (INCI), the wine lees will be identified as Saccharomyces/Grape Lees Ferment extract, and it is obtained under the lyophilization of the extract recovered after sonication. The others ingredients used in the formulations are listen in Table 3. Formulations were selected to improve product consistency, strength, texture and functionality.

Table 4 indicates the list of ingredients (INCI names) and procedures used for the preparation of the cosmetic products. When homogenization was necessary, a T25 Ultra-Turrax^®^ (IKA™, Staufen, Germany) was used (the speed is indicated time to time). The stirring was performed by an IKA™ EUROSTAR 20 High Speed Digital Overhead Stirrer.

### 2.8. Physicochemical Characterization of Formulations

The formulations were analyzed for pH with a pH 60 Violab bench pHmeter equipped with the electrode XS Sensor Flow S7 Ag/AgCl, with an internal solution (electrolyte) of KCl 3M. The density of the cleansing powder was determined by measuring the volume in a graduated cylinder of the accurately weighted powder. The evaluation of the stability under accelerated conditions was performed placing the formulations at both 4 and 40 °C alternatively, under triplicate cycles. All the formulations were also tested for a long-term stability at room conditions for 1 year. The formulations were considered to have passed the test if no changes in organoleptic characteristics occurred (color, odor).

### 2.9. Challenge Test

In spite of the fact that all formulations are solid cosmetic formulations where a minimum amount of water has been used and only remains in small amount in the final product, the evaluation of possible microbial contamination of the products under use is necessary, which is why a challenge test was performed according to Regulation CE 1223/2009. The challenge test was performed according to the method ISO 11930:2012 Cosmetics–Microbiology–Evaluation of antimicrobial protection of a cosmetic product [35]. The antimicrobial activity was screened against *Pseudomonas aeruginosa* ATCC 9027, *Staphylococcus aureus* ATCC 6538, *Candida albicans* ATCC 10231, *Aspergillus brasiliensis* ATCC 16404 and *Escherichia coli* ATCC 87394. *Pseudomonas aeruginosa*, *Staphylococcus aureus* and *Escherichia coli* were incubated for 48–72 h at 30 °C on Tryptic Soy Agar (TSA); *Candida albicans* was incubated for 48–72 h at 30 °C on Sabouraud Dextrose Agar (SDA); and *Aspergillus brasiliensis* was incubated for 72–120 h at 22.5°C on Potato Dextrose Agar (PDA). The media TSA, SDA and PSA were from Sigma Aldrich, Stenheim, Germany. Initial inoculi were 300,000, 480,000, 700,000, 41,000 and 9000 UFC/g for *Pseudomonas aeruginosa*, *Staphylococcus aureus*, *Escherichia coli*, *Candida albicans* and *Aspergillus brasiliensis*, respectively. The test was followed for 28 days.

### 2.10. Local Compatibility Test with Human Skin (Irritant Potential)

The test for the evaluation of the compatibility (irritant potential) of the cosmetic formulations with human skin was performed under normal conditions according to the Helsinki Declaration (64th WMA General Assembly, Fortaleza, Brazil, October 2013) and to the COLIPA guidelines [36]. A panel of 10 volunteers (age 28 ± 3 years old) of female and male gender was used. The products were left in contact with human skin for 48 h (model Curatest^®^ F, adhesive strips for patch test, Lohmann and Rauscher International, Rengsdorf, Germany) in a sufficient amount to fill a 1 cm**^2^** test disk. The assessment was made with the comparison with a negative control. The reactions of the skin were evaluated 15 min after patch removal, and again after 24 h, according to defined parameters (erythema, desquamation, oedema and vesicles). The tests were performed in single blind mode, under the directions of a medical doctor certified in dermatology.

### 2.11. Measurement of the Trans Epidermal Water Loss (TEWL)

The measurements of trans epidermal water loss (TEWL) for the evaluation of the efficiency of the skin barrier were conducted with a VapoMeter^®^ (Delfin Technologies, Kuopio, Finland) that evaluates humidity with a closed chamber unaffected by ambient airflows. The increase of relative humidity (RH) was measured by the sensor and the evaporation rate value (g/m**^2^**h) was automatically calculated from the RH increase. The measurements were conducted after the application of the formulations on the forearm divided in six parties of the 20 volunteers, in a room with controlled humidity and temperature (57.2% and 23.4 °C). Five measurements for each product were taken: the first before the application of the products, and 60, 120, 180 and 240 min after rinsing the products. The two masks were left 15 min on the skin after the application. The measuring time was around 10 s.

### 2.12. Measurement of the Sebometry

The amount of sebum (µg/cm^2^) on the skin was measured using SebumScale (Delfin Technologies, Kuopio, Finland) in a room with controlled temperature and humidity of 23 °C and 54%. A disposable quartz crystal sensor was placed on the skin for some seconds to absorb the sebum. The equipment measures the mass of the collected sebum analyzing the changes on the quartz crystal resonance frequency. Measurements were made after the application on the forehead of the 20 volunteers of the solid facial cleanser, cleansing powder and two masks, avoiding the make-up remover, which represents a preliminary step during skin care. Products were left on the skin for 15 min. Five measurements for each product were taken: the first before the application of the products and after 30, 60 and 120 min after rinsing the products. The measuring time was around 15 s.

## 3. Results and Discussions

### 3.1. Microbiological Analysis

#### 3.1.1. Analysis of the Microbiome in the Lees

Since the microbiological variability of the wine lees under study is unknown and potentially high, a Next Generation Sequencing approach was used to obtain information on the microbiome of the sample. This is an inexpensive and fast approach that can guide subsequent steps to characterize the viable fraction of microbes with a focus on potential pathogens. The 16S rDNA NGS analysis was used to evaluate the bacterial diversity in the lees, while the 18S ITS NGS analysis was used for the eukaryotic cells, which are expected to be mainly represented by yeasts.

**Figure 2:**
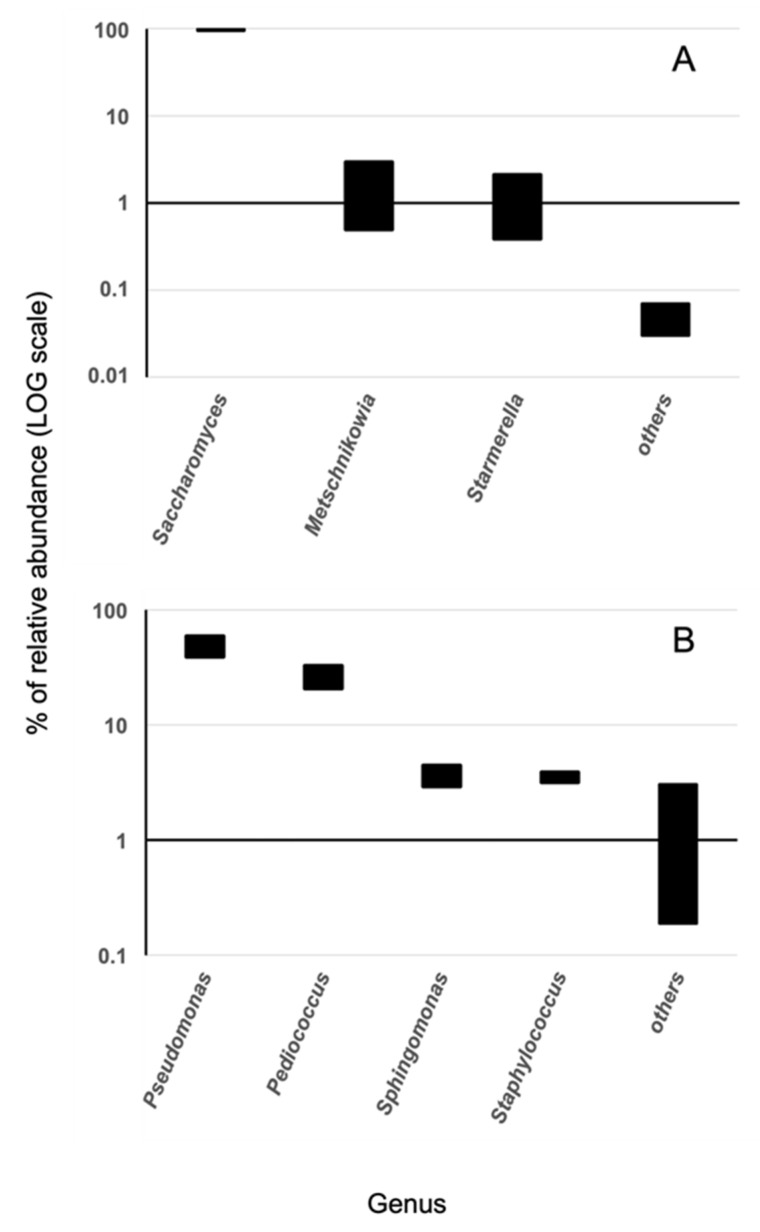
Relative abundance of genus represented in the lees sample. (**A**): Fungal diversity by 18S ITS NGS analysis. (**B**): bacterial diversity by 16S rDNA NGS analysis. The group “others” comprises low represented genus (<3% relative abundance). Bars represent ranges of abundance obtained by two measurements.

The results are shown in Figure 2. As expected, the *Saccharomyces* genus is the most represented (95–99%), and the associated ITS sequences found a match at the level of species to the *Saccharomyces cerevisiae*, which is essential and well-known for driving fermentation processes in the winery. In turn, this evidence is supported by DLS and SEM analysis, which revealed cellular bodies having shape and size compatible with yeast cells. This evidence is quite important in the context of the present work, as the main claim here is the major contribution of the cells and cellular components of the *Saccharomyces cerevisiae* contained in lees for the development of cosmetic products.

It is worth noting the presence of other non-*Saccharomyces* yeasts in the lees, which may be significant from an oenological point of view, namely the genera *Starmerella* and *Metschnikowia* (Figure 2). The former has been resolved at the species level as *Starmerella bacillaris* (synonym *Candida zemplinina*) [37]**,** and is often used in combination with Saccharomyces in alcoholic fermentation to increase the quality of the process [38]. The latter corresponded to the species *M. pulcherrima*, which has a strong biocontrol activity against other undesirable yeast species possibly present in winemaking [38].

The bacterial diversity was featured by the prevalence of species belonging to the genus *Pseudomonas* (49.1%) and *Pediococcus* (26.6%), and to a lesser extent *Sphingomonas* (3.7%) and *Staphylococcus* (3.5%). The *Pseudomonas* genus is ubiquitous in the environment and includes more than one hundred species. Few species are human opportunistic pathogens such as *P. aeruginosa*. However, NGS analysis did not assign it in the lees sample [39]. Various species belonging to the genus *Pediococcus* are utilized in industrial fermentations of foods and silage, are considered a probiotic and may be present during the production of wines. Their presence may be desirable both for biocontrol and to add a special taste to the final product [40]. Some species have been rarely associated with human infections, but only in immunocompromised patients [41]. The genus *Staphylococcus* is mainly associated with animals, and it includes many different species, some of which are responsible for diseases in humans [42]. *Sphingomonas* is notoriously very resistant to various stress conditions and can be found in various environments [41]. Different genera were among the less prevalent (<3%). *Faecalibacterium* (e.g., *F. prausnitzii*) is a mesophilic bacterium usually isolated from human feces [43]. It is considered a marker of healthy intestinal microbiota and non-pathogenic. The genus *Tatumella* spp., typical of fruit and soil, has been associated with wine production [41]. Species belonging to the genus *Acinetobacter* and *Gluconacetobacter* are important soil bacteria [44]. *Acinetobacter* spp. are environmental, and only a few species are associated with nosocomial infectious diseases in immunocompromised patients. Genus *Variovorax* spp. has been isolated in a diverse range of environments [45]. *Enhydrobacter* genera have not been associated with any type of disease. The name genus *Burkholderia* refers to a group of Gram-negative bacteria characterized by species isolated from humans, animals and plants [46]. Only a few species are potentially pathogenic. Being ubiquitous in nature and a common airborne organism, *Methylobacterium* spp. is found in a wide variety of environmental, industrial and clinical environments [27,47]. Additionally, *Micrococcus* occurs in a wide range of environments, including water, dust and soil; this genus is generally thought to be saprotrophic or commensal, though it can be an opportunistic pathogen, particularly in hosts with compromised immune systems, such as HIV patients [48]. *Natronobacillus* is a bacterial genus that can be found in soils [49], while *Alistipes* is primarily isolated from the intestines of patients with appendicitis. Bacteria of the genus *Bacteroides* are commensals of the intestinal and urogenital tracts of humans and other animals; they are found in the oral cavity and are the most numerous bacteria in human feces [50].

Based on this NGS analysis, we planned the analysis of the viable bacteria content of the lees to exclude the presence of undesired mesophilic species according to the ISO 17516 standard [27].

#### 3.1.2. Count of Mesophilic Bacteria, Anaerobes, and Fungi

Like all products on the market, cosmetic products must meet certain requirements to ensure product safety and protect the health of consumers. The microbiological quality of cosmetic products is regulated by the ISO 17516 standard that involves total aerobic mesophile counting and the search for specific microorganisms, including Enterobacteriaceae, *Pseudomonas* spp., *S. aureus* and *C. albicans* [51]. Microbial contamination may derive from one or more sources, such as raw material [52].

The total count of viable mesophiles was 5 × 102 CFU/mL of lees. This value is below the maximum limit indicated by requirements for a finished cosmetic product, which is set to 103 CFU/mL [51]. Considering that the quantity of lees used for formulation would certainly be a fraction of the total amount of the final product, the results are promising in terms of microbiological safety related to the source material. Moreover, the final product usually contains actives to control microbial growth, which could evenly decrease microbial viability in the final product. Interestingly, no viable fungi nor anaerobes were found. The first result would indicate that the fraction of yeast cells composing the lees, which is predominant, is not viable.

#### 3.1.3. Growth of Isolates on Selective Media

Sixty-four bacterial isolates from the plates used to make the total count of viable mesophiles were randomly selected and subcultured onto MCA, PCA and MSA, which are selective and differential. MSA allows the growth of halotolerant bacteria, while the color change from purple to yellow is visible only when bacteria can ferment mannitol. MCA medium is used to favor the growth of lactose fermenting Enterobacteriaceae and is selective, thanks to the presence of crystal violet and bile salts. PCA facilitates the growth of bacteria resistant to cetrimide, such as *Pseudomonas* spp. No bacterial growth occurred on PCA and MCA. This result indicated that the sample is most probably devoid of any viable *Enterobacteriaceae* and *Pseudomonas*. On the contrary, a clear growth was visible on MSA plates. Eighty-three per cent of them were able to ferment mannitol. This would be an indication of the putative presence of *Staphylococcus aureus*. Although growth with a color change is also possible for some other bacterial species—and the lees sample cannot be directly associated with a human sample, so that contamination with *S. aureus* is unlikely—the results suggest further microbiological analysis to confirm or exclude the presence of pathogens considered by ISO 17516 [27].

#### 3.1.4. Identification of Mesophiles Isolates Grown onto MSA by 16S rDNA Analysis

**Figure 3:**
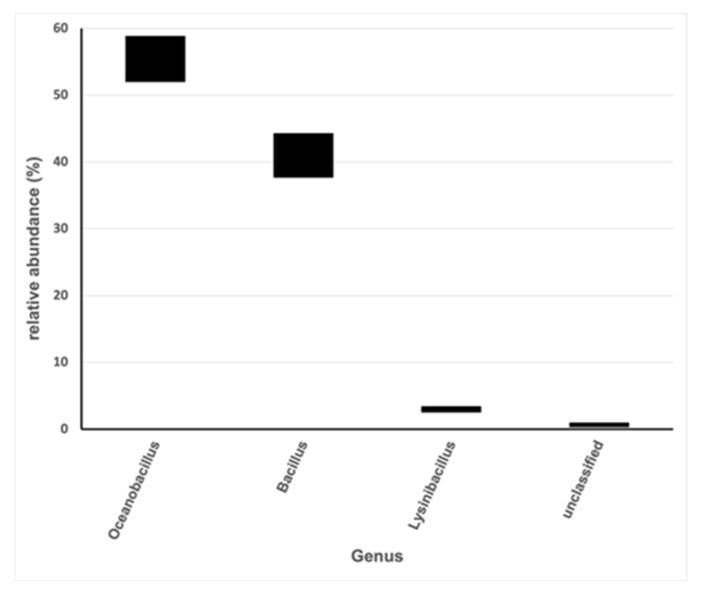
Relative abundance of genus represented in the pool of isolates from viable cells counting experiments. Bacterial diversity was assessed by 16S rDNA NGS analysis. The group “others” comprises low represented genus (<1% relative abundance). Bars represent ranges of abundance obtained by determinations made on two independent pooled samples.

Isolates from plates were pooled together. Total DNA from the pooled cells was extracted and the identification was determined through 16S rDNA analysis by NGS. Bacterial genera found in the sample shown in Figure 3 belong to the phylum Firmicutes and are environmental [53,54,55,56,57]. They are Gram-positive, halotolerant, aerobic, or facultative anaerobic and spore-forming. Their general growth characteristics are summarized in Table 5 and correlate with those used to isolate and differentially cultivate them in this study.

The color change onto MSA is explained by the fact that most of the identified bacterial species have the enzymatic pathway enabling them to ferment mannitol. Overall, the results allow us to exclude, under these experimental conditions, a measurable contamination by potentially pathogenic viable bacterial cell. However, it is important to consider that this analysis pertained to one single batch of lees. A more extensive analysis considering a significant number of batches would give a reliable evaluation of the general microbial diversity of the Belisario lees (and lees from other wines), which, in turn, would help in guiding the safe inclusion of this important and valuable byproduct in cosmetics.

### 3.2. Optimization of Ultrasound-Assisted Extraction Method for the Yeast Cells Breakdown in Water and Evaluation of the Properties of the Aqueous Extract

During the present study, a systematic optimization of the sonication conditions, such as, for example, the dilution, the percentage of amplitude or the sonication time, has been performed in order to reduce both energy and time necessary to break up the cells.

**Figure 4:**
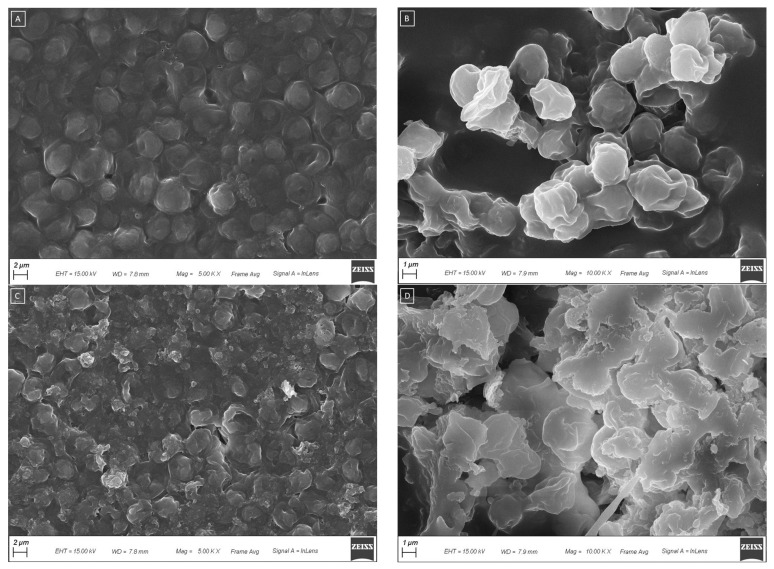
SEM images of (**A**) NWL (5.00K X); (**B**) lyophilized NWL sample (10.00K X); (**C**) SWL (5.00K X); (**D**) and lyophilized SWL (10.00K X).

**Figure 5:**
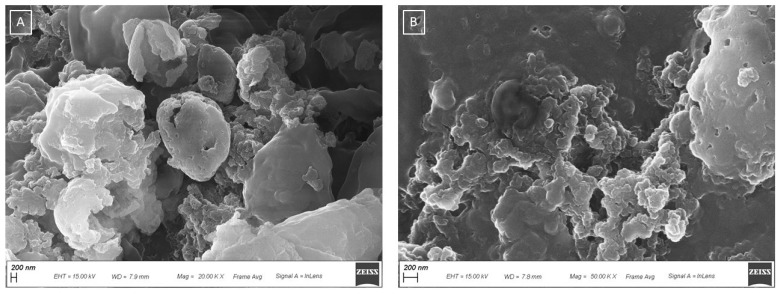
SEM images of lyophilized SWL showing yeast cells breakdown and cellular aggregation. SEM of (**A**) SWL (20.00K X) and (**B**) SWL (50.00K X).

The optimization of the extraction method has been performed by comparing the samples produced under different sonication conditions under both optical (prevalently used routinely) and scanning electron microscopy, and DLS analysis, and verifying the capacity of each specific treatment to favor the yeast breakdown. The pictures obtained with SEM were compared with images of yeast cells found in the literature [61]. Figure 4 shows the aspect of NWL (Figure 4A,B) and the SWL (Figure 4C,D), obtained under the optimized conditions, observed under SEM. The cells of NWL appear to be separated from each other, even if they do not exhibit a plump and oval-shaped morphology with a smooth envelope, thus suggesting they are not vital and lysed (Figure 4A,B). In contrast to the NWL, after sonication, the shape of the cells showed various degrees of deformation, and some cells totally collapsed (Figure 4C,D and Figure 5). The sonicated sample exhibits a clear cells breakdown (Figure 5A), which leads to cellular aggregation (Figure 5B). As described in the Methods section, the optimized sonication occurred for 5 min of sonication of a 1:10 water diluted suspension, with a constant sonication frequency of 20 kHz, an amplitude of 99% and a 19-mm probe with a continuous sonication mode. Lower sonication amplitude or a pulsated sonication mode or less diluted samples requested longer time for cell breaking up. The percentage yield of dry weight was calculated for both NWL and SWL. A yield of 4.9% and 5.6% were obtained for NWL and SWL, respectively. The weights of the supernatant of both NWL (3.5 g) and SWL (2.8 g) from the total 10 g of starting material were calculated. Considering the amount of supernatant calculated in the starting material and the water added for dilution (90 g per sample), a dry weight concentration of 2.1 mg/ and 1.5 mg/mL was obtained for NWL and SWL, respectively.

**Figure 6:**
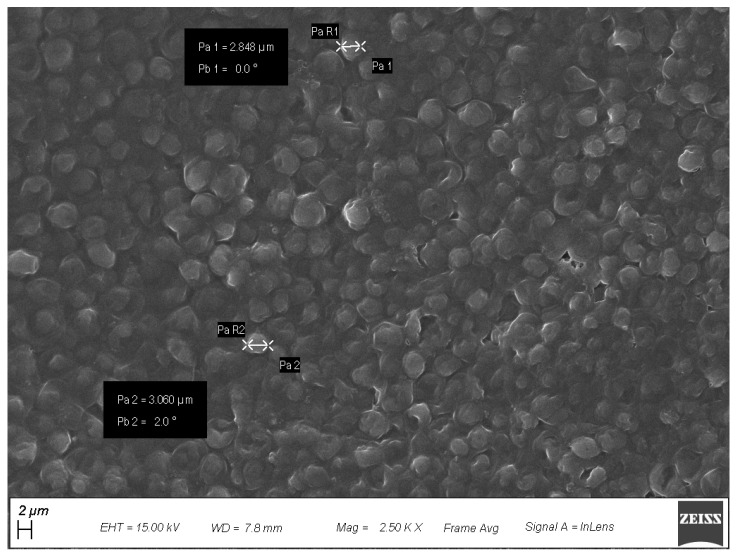
Yeast cells size measurement by SEM analysis of NWLs.

The diameter of some cells was measured (Figure 6), which fell in the range of 2848–3000 nm, in agreement with the 1000–10,000 nm range reported in the literature for yeast cells [62].

The breakdown and perforation of the cell wall has been described for several different cells. In a recent study, the effects of ultrasound on yeast cells are related to the breakdown of the cell walls, disruption and thinning of cell membranes [59]. The mechanism of action by which ultrasound disrupts cells is called cavitation: when sound waves travel through the liquid system, they generate cycles of particle compression and expansion, and many cavities are formed thereof; these cavities are filled with air or vapor [61], producing strong implosions and explosions in the medium with a major increase in temperature and pressure, which could be responsible for cell wall perforation with subsequent rupture.

To further confirm the efficiency of sonication in breaking down of the yeast cells, the yeast size was determined by DLS analysis, and the results are shown in Table 6. The largest yeast size was observed for the sample (NWL0) diluted 1:10 in water, not subjected to any treatment (neither lyophilization of sonication). Once lyophilized (NWL1), the cell size decreased because of the water loss, and cells appeared with a wrinkled cell wall (Figure 4B). Sonication strongly affects the cell size for the samples (SWL0 and SWL1), which is the lowest among all the samples, and the lees’ samples subjected to sonication, but not lyophilized has a smaller value. Therefore, disruption of the yeast cells through sonication could lead to an average reduction in yeast cell size. The SWL1, resuspended in water after lyophilization to allow the DLS analysis, exhibited a higher size than the sample SWL0, which was not lyophilized. This is probably due to the fact that the lyophilized sample has a strong affinity for water, and tends to absorb a large amount of water that allows for a swelling. This same phenomenon cannot be described for the NWL, because the sample redispersed in water after the lyophilization NWL1 is smaller than the NWL0. Independently of the treatment, the diameter of the yeast cells fell within the 1000–10,000 nm range found in the literature [62], which confirms the results obtained by SEM analysis.

Protein analysis was performed to confirm the efficiency of sonication in allowing the release of both the cytoplasmic proteins, and the one associated with the yeast cell wall. The total protein content for the supernatant of the NWL was 0.117 ± 0.001 mg/mL, while the supernatant of the SWL was 0.284 ± 0.009 mg/mL. This result indicates the substantial disruption of the yeast cell that causes the release of cell proteins. Prolonging sonication can cause significant protein denaturation resulting from the final temperature increase driven by insufficient cooling [63]. In this case, an increase in protein content occurred in the supernatant of the lees subjected to the sonication process, indicating that sonication time and power are appropriate.

The element analysis was performed to evaluate the safety of wine lees regarding to the heavy metals content, while valorizing the wine lees regarding the benefits for the skin due to the presence of macro and microelements. The ICP-MS results of the total element content are reported in Table 7, and a certain difference in concentration for most of the elements between the supernatant of NWL and SWL can be highlighted. For some elements, the concentration is higher in the supernatant of SWL (Li, B, Na, K, Ca, Mn, Zn, Rb, Sr, Ba), and this could be due to the escape of the elements in saline form from the yeast cells after breaking. Contrarily, Mg, P, Cu and Fe are found in higher concentration in the supernatant of the NWL with respect to the sonicated ones, and it could depend on the binding with the biological tissues, after being released from the yeast cells.

Concerning the limits in heavy metals, the Regulation (EC) 1223/2009 indicates that heavy metals are technically unavoidable in traces, and the absence of limit values, requires a case-by-case assessment [55,64]. The absence of quantitative limits has led some Member State authorities, such as the ‘Ministry of Health’, to establish guide values to be respected. In Italy, the “Istituto Superiore di Sanità” (ISS) established maximum limits as “technically unavoidable traces” [64]. The concentration of heavy metals in lees can be assessed after optimizing the method for the element’s quantification. Table 7 shows the content in chromium that can be considered safe. Other heavy metals were not detected.

The antioxidant capacity of NWL and SWL was evaluated. The results, reported in Table 8, show that all the assays share the same trend. NWL showed the lowest antioxidant capacity, while the sonication of the lees favored the release of molecules with an antioxidant capacity. In fact, all the cells of *Saccharomyces Cerevisiae*, present in the lees, have a natural antioxidant defense system composed of small molecules like glutathione (GSH) and ascorbic acid, and enzymes like superoxide dismutases (SODs), catalases, glutathione peroxidases (GPXs) and peroxiredoxins (Prxs) [65] that can be released after the breakup of the cells wall [66].

Wine lees is known for its high content in lipids, which include the presence of squalene [17]. In the lipidic extract of Matelica’s Verdicchio obtained with ultrasound, the squalene content detected by using HPLC-DAD is 2.272 mg/g. It can be considered an important vegetable and ethical source of squalene comparable to olive oils, with the advantage f being recovered from waste [67]. The use of wine lees as a rich source of this bioactive compound also represents a promising approach for a cost-effective, environmentally friendly investment by the winery industry and an alternative and sustainable source of ingredients for the cosmetic sector.

### 3.3. In Vitro Metabolic Activity and Cytotoxicity Assays

**Figure 7:**
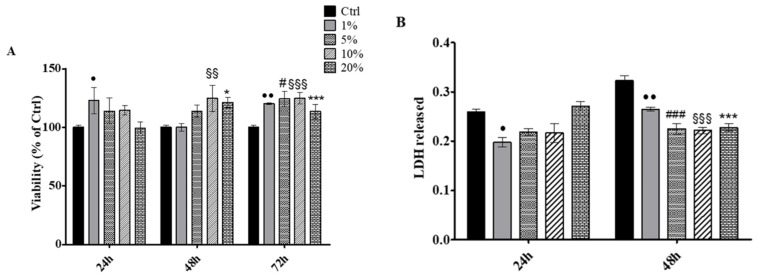
(**A**) MTT assay performed on Hacat keratinocytes treated for 24 h, 48 h and 72 h with SWLs in percentages ranging from 1 to 20%. Metabolic activity was normalized to control cells treated with DMEM culture medium. The most representative of three separate experiments is shown. Data are presented as the mean ± standard deviation. • vs. Ctrl 24 h *p* < 0.01, * vs. Ctrl 48 h *p* < 0.01, §§ vs. Ctrl 48 h *p* < 0.001, §§§, # vs. Ctrl 72 h *p* < 0.01, •• vs. Ctrl 72 h *p* < 0.001, §§§, *** vs. Ctrl 72 h *p* < 0.0001. (**B**) LDH release from HaCat keratinocytes treated for 24 and 48 h with SWLs in percentages ranging from 1 to 20%. The most representative of three separate experiments is shown. Data are presented as the mean ± standard deviation. • vs. Ctrl 24 h *p* < 0.01, •• vs. Ctrl 48 h *p* < 0.001, ###, §§§, *** vs. Ctrl 48 h *p* < 0.0001.

First, SWLs were administered to HaCat keratinocytes cell line in percentages ranging from 1 to 20% for 24, 48 and 72 h. The metabolic activity was read by means of MTT test. After 24 h of treatment, a statistically significant increase in cell viability was recorded in the presence of 1% SWLs respect to untreated cells (Ctrl); after 48 h the metabolic activity was significantly augmented with 10 and 20% SWLs compared to the control, while, after 72 h of treatment, the viability of HaCat exposed to all the SWLs tested percentages (1, 5, 10 and 20%) appeared significantly increased with respect to the control (Figure 7A). Then, in order to evaluate the cytotoxicity level within the culture medium, a LDH assay, after 24 and 48 h of treatment, was carried out. After 24 h of SWLs exposure, a statistically significant reduction of released LDH was recorded in HaCat treated with 1% SWLs with respect to untreated cells, whereas, after 48 h, all the administered SWLs per-centages led to a significant decrease in LDH spread within the culture medium with respect to the control cells (Figure 7B).

Taken together, these data lead us to assume that SWLs definitely show a safe and beneficial profile on keratinocytes by promoting, on the one hand, an appreciable increase in cell viability, and, on the other hand, by significantly reducing the cytotoxicity level.

**Figure 8:**
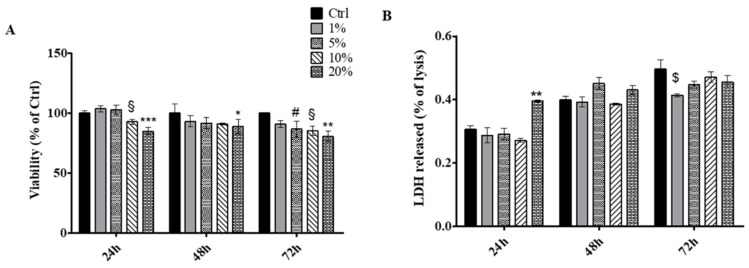
(**A**) MTT assay performed on HGFs treated for 24 h, 48 h and 72 h with SWLs in per-centages ranging from 1 to 20%. Metabolic activity was normalized to control cells treated with DMEM culture medium. The most representative of three separate experiments is shown. Data are presented as the mean ± standard deviation. § vs. Ctrl 24 h *p* < 0.01, *** vs. Ctrl 24 h *p* < 0.0001, * vs. Ctrl 48 h *p* < 0.001, #, § vs. Ctrl 72 h *p* < 0.01, ** vs. Ctrl 72 h *p* < 0.001. (**B**) LDH release from HGFs treated for 24 and 48 h with SWLs in percentages ranging from 1 to 20% 24 h and 48 h after treatment with wine lees. The most representative of three separate experiments is shown. Data are presented as the mean ± standard deviation. ** vs Ctrl 24 h *p* < 0.001, $ vs. Ctrl 48 h *p* < 0.01.

Second, SWLs were tested on connective tissue cells represented by primary HGFs. The metabolic activity and cytotoxicity level were measured. An MTT test was carried out after 24, 48 and 72 h administering grape lees at 1, 5, 10 and 20 % to HGFs. After 24 h of SWLs exposure, a statistically significant reduction of cell viability was evidenced in HGFs treated with 10 and 20% SWLs with respect to untreated cells. After 48 h, the cell viability level appeared significantly reduced with respect to the control, when 20% SWLs was administered, while, after 72 h, HGFs exposed to 5, 10 and 20% SWLs disclose a statistically significant reduction of cell viability with respect to untreated cells (Figure 8A). The obtained results underline the fact that very high SWL percentages (10 and 20%) could lightly affect HGF viability, thus suggesting, as already evidenced elsewhere [34], on the importance of continuing SWL biological evaluations with lower concentrations (1 and 5%), in order to totally preserve HGF viability. To conclude, LDH release within the culture medium was evaluated, finding, after 24 h of treatment, a significant augmentation of released LDH% in 20%-treated HGFs with respect to untreated cells, while, after 72 h, a statistically significant reduction of released LDH% was disclosed in HGFs exposed to 1% SWls (Figure 8B).

### 3.4. Cosmetic Products

Due to the properties of the SWLs, five new solid cosmetic products were formulated. All products were formulated to create an attractive and innovative beauty routine with the least amount of water and using natural ingredients. The use of minimal amounts of water supports product preservation (solid cosmetics last longer than liquid) and plays within the respect of water waste. Solid products can be easily transported, and are suitable for travelling. They do not require plastic packaging, as they can be easily contained in paper packaging. Most of the products are formulated in a convenient size to ensure easy application. The general idea is to follow the current trend of the world cosmetics market, with the concept of sustainability.

The addition of Saccharomyces/Grape Lees Ferment into the formulations, thanks to its antioxidant properties and cell viability, corresponds with the skin protective action of the cosmetic products; the content of polyphenols, proteins, squalene and microminerals that can be found in an appreciable quantity in the SWL offer a precious cosmetic ingredient with potential hydrating properties. Finally, the complexity in the microbial composition, within its safe profile, which could correspond with benefits for the skin microbiota, completes the cosmetic interest for SWL. The Saccharomyces/Grape Lees Ferment used in all the formulations was obtained after sonication and the lyophilization of the whole extract, which is composed of both the liquid and the solid fractions.

#### 3.4.1. General Characteristics of the Cosmetic Products

The general characteristics of the five cosmetic formulations are reported in Table 9. All the formulations were in a pH range between 5.8 and 6.8, allowing for care of the skin’s pH. The density of the cleansing powder is 380 g/L, and is the only product not formulated for being monoportion.

All the formulations were stable under both accelerated and long-term conditions. No physicochemical instability phenomena were observed, such as changes in smell or color. All the formulations passed the challenge test, and they were also well tolerated by normal human skin, with a mean irritation index of 0, observed 15 min and 24 h after application, for all the tested volunteers.

#### 3.4.2. Evaluation of Clinical Efficacy

**Figure 9:**
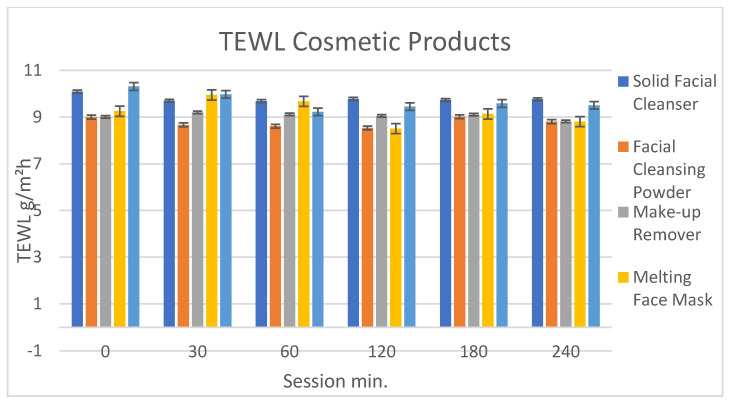
TEWL measurement before the application of the cosmetic products and 30, 60, 120, 180 and 240 min after the removal.

Trans epidermal water loss (TEWL) is an indication for an alteration in the capacity of the skin to contrast a water loss from the more external skin layers, and in general it increases as a consequence of a damaged skin. Apart from pathological conditions, the use of aggressive cleansers can lead to a significant increase in the TEWL. None of the five formulations showed a change in TEWL with respect to the starting values. Small changes visible on the graph (Figure 9) are not statistically significant (significance *p* < 0.01).

#### 3.4.3. Measurements of the Sebometry

Sebometry is a test that evaluates the capacity of a cosmetic cleanser product to remove the sebum layer during a cleansing procedure. Sebum removal can occur mechanically or most frequently thanks to the presence in the formulation of surfactants or absorbing agents.

**Figure 10:**
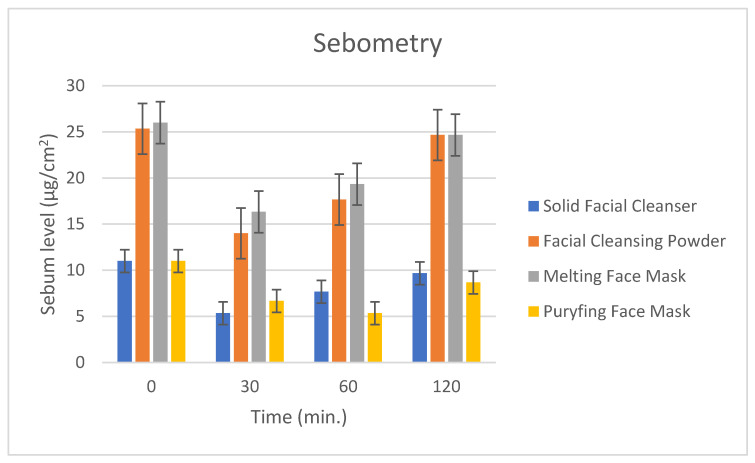
Skin sebum measurement before the application of the cosmetic products, and after 30, 60 and 120 min after the removal of that.

**Figure 11:**
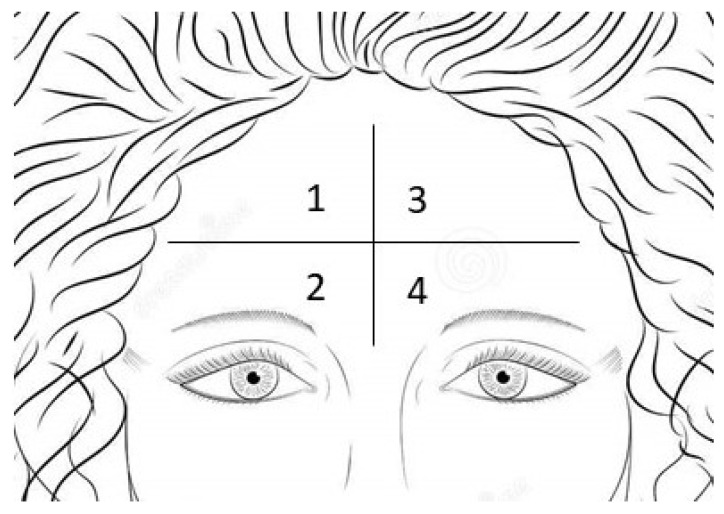
Forehead division for measurements of sebometry. Each product was applied in one of the four areas of the forehead.

The results are reported in Figure 10. To better interpret the results, it is important to highlight that the differences in the time 0 (before the application of the cosmetic products) depend on the different application points of the forehead (Figure 11). An immediate statistically significant (*p* < 0.01) decrease in the sebum level was observed after 30 min of the treatment for all the products. This means that the application of these products allowed for the removal of the sebum from the skin surface, which is appropriate for such a product, thanks to the presence of ingredients such as light surfactants. The sebum content tends to be restored 120 min after treatment for the solid facial cleanser, the facial cleansing powder and the melting mask. The purifying face mask that contains higher amounts of Saccharomyces/Grape Lees Ferment did not restore the initial sebum level during the testing time, making it possible to support a higher purifying capacity of this ingredient.

## 4. Conclusions

This study is intended to encourage the exploitation of sonicated wine lees, a wine waste that is not yet exploited as a beneficial ingredient in the cosmetic industry. The results of the present study represent of a good example of the exploitation of waste from food industry from an upcycling perspective.

The results obtained in this study for this specific batch cannot be directly extended to other batches or types of lees, and thus a batch-to-batch characterization will be necessary for product marketing.

## Figures and Tables

**Table 1:** Incubation conditions of the plates inoculated with wine lees samples (NWLs).

Medium	Sample	Incubation Temperature (°C)	Atmospheric Conditions
TSA	F1:10	37	Aerobiosis
TSA	F1:100	37	Aerobiosis
TSA	F1:10	Room temperature	Aerobiosis
TSA	F1:100	Room temperature	Aerobiosis
SDA	F1:10	37	Aerobiosis
SDA	F1:100	37	Aerobiosis
SDA + chloramphenicol	F1:10	37	Aerobiosis
SDA+ chloramphenicol	F1:100	37	Aerobiosis
MRS	F1:10	Room temperature	Aerobiosis
MRS	F1:100	Room temperature	Aerobiosis
MRS	F1:10	Room temperature	Anaerobiosis
MRS	F1:100	Room temperature	Anaerobiosis

**Table 2:** Microwave digestor program.

Step	1	2	3
T (°C)	150	190	50
P (Bar)	36	36	0
POWER (%)	70	90	0
RAMP (Min)	5	5	1
Step (Min)	10	20	20

**Table 3:** Ingredients used in the formulation of cosmetic products, their function and the supplier.

Commercial name	Ingredients (INCI)	Supplier
TEGO^®^ Betain CK D MB	Cocamidopropyl BetaineSodium Chloride, Aqua	Evonik, Essen, Germany
	Stearic acid	Acef, Fiorenzuola d’Arda (PC), Italy
	Cetearyl alcol	
	Theobroma Cacao Seed Butter	
	Butyrospermum Parkii Butter	
	Citric acid	
	Glycerin	
	Kaolin	
	Zinc oxide	
	Helianthus Annuus Seed Oil	
	Cera Alba	
	Hydrogenated castor oil	
	Oryza Sativa Starch	
	Argania Spinosa Kernel Oil	
	Oryza Sativa Bran Oil	
ACNIBIO PE 9010	Phenoxyethanol, ethylhexylglycerin	
GLDA Chelating	Aqua, Tetrasodium glutamate diacetate, Sodium hydroxide	
	Butylene Glycol	
	Oryza Sativa Bran Oil	
	Sodium Stearate	
	Thocopheryl Acetate	Basf, Ludwigshafen, Germany
	Sodium Coco-Sulfate	
	Glyceryl Stearate	
	Betaine	
	Erylite	Eurotrading, Civitanova Marche (MC), Italy
	Cocos nucifera oil	Esperis, Milan, Italy
	Copernicia Cerifera Cera	Natura Tec, Louis Lépine Fréjus, France
Turkey Red Oil	Castor Oil Sulfated, Sodium Salt, Aqua	Zschimmer & Schwarz, Lahnstein, Germany
PROTELAN ENS	Stearic acid, Sodium lauroyl glutamate, Cetearyl alcohol, Glyceryl stearate	
	Hydrogenated Coco-glycerides	Farmalabor, Canosa di Puglia (BT), Italy
	Polyglyceryl-10 Caprylate	Bregaglio, Biassono (MB), Italy
SORBOSIL™ AC 36	Hydrated silica, Aqua	
	Cetyl Alcohol	
	Sodium Cocoyl Glutamate	Prodotti Gianni, Milan, Italy
	Hydroxyethylcellulose	Vevy Europe, Genova, Italy

**Table 4:** List of ingredients and preparation of cosmetic formulations.

Cosmetic Formulation/ Consistency	List of Ingredients According to the International Nomenclature Cosmetic Ingredients (INCI)	Method of Preparation
Facial cleanser Solid	Sodium Coco-sulfate, Oryza Sativa Starch, Sodium Cocoyl Glutamate, Cetearyl Alcohol, Glyceryl Stearate, Hydrogenated Coco-glycerides, Saccharomyces/Grape Lees Ferment Extract, Butyrospermum Parkii Butter, Betaine, Hydrogenated Coco—glycerides, Aqua, Theobroma Cacao Seed Butter, Tocopheryl Acetate, Tocopherol, Parfum, Citric Acid	The lipidic phase A was weighed and heated in a water bath up to 85 °C until complete melting. Vitamin E was added to the Phase A once the latter was removed from the water bath and allowed to cool under stirring. Sodium coco-sulfate, oryza sativa starch and the Saccharomyces/Grape Lees Ferment were weighed and homogenized to the lipidic phase at 3500 rpm to obtain a pasty texture. Citric acid was weighed and stirred until complete solubilization in water and added to the paste to reach the desired pH. Perfume was added as the last step.
Facial cleansing Powder	Oryza Sativa Starch, Saccharomyces/Grape Lees Ferment Extract, Oryza Sativa Bran Oil, Sodium Cocoyl Glutamate, Erylite, Silica, Betaine, Cocamidopropyl Betaine, Sodium Chloride, Parfum, Aqua	The oil was added drop by drop to be adsorbed into the weighed starch by using a pestle and a mortar. Thus, first Saccharomyces/Grape Lees Ferment, and then betaine, silica and erylite were added and mixed with a mortar to obtain a homogeneous powder. Perfume was added as the last step.
Make-up remover Solid	Helianthus Annuus Seed Oil, Hydrogenated Coco-glycerides, Cera Alba, Polyglyceryl-10 Caprylate, Saccharomyces/Grape Lees FermentExtract, Theobroma Cacao Seed Butter, Castor Oil Sulfated, Cocos Nucifera Oil, Butyrospermum Parkii Butter, Hydrogenated Castor Oil, Sodium Salt, Tocopheryl Acetate, Parfum, Aqua.	The lipidic phase was weighed and heated in a water bath up to 90 °C until complete melting. Vitamin E was added after the removal from the water bath of the mixture and allowed to cool under stirring. Saccharomyces/Grape Lees Ferment was dispersed in water and added to sulfated castor oil s, heated up to 40 °C under stirring to promote the formation of the mixture and added to the lipidic phase. Perfume was added as the last step.
Melting face mask Solid	Cocos Nucifera Oil, Kaolin, Saccharomyces/Grape Lees FermentExtract, Butyrospermum Parkii Butter, Copernicia Cerifera Cera, Castor Oil Sulfated, Sodium Salt, Zinc Oxide, Tocopheryl acetate, Parfum, Aqua.	Cocos nucifera oil, butyrospermum parkii butter and cera carnauba were weighed and heated up to 80 °C for 30 min under stirring with kaolin and zinc oxide. After removing the ingredients from the water bath, vitamin E was added. Saccharomyces/Grape Lees Ferment was dispersed in water and added to sulfated castor oil, heated up to 40 °C under stirring to promote the formation of the mixture and added to the other ingredients. Perfume was added as the last step.
Purifying face mask Solid	Aqua, Kaolin, Glycerin, Saccharomyces/Grape Lees FermentExtract, Butyrospermum Parkii Butter, Sodium Stearate, Butylene Glycol, Glyceryl Stearate, Cetearyl alcohol, Cera Alba, Stearic Acid, Sodium Lauroyl Glutamate, Tocopheryl Acetate, Phenoxyethanol, Hydroxyethylcellulose, Parfum, Ethylhexylglycerin, Tetrasodium Glutamate Diacetate, Sodium Hydroxide.	Phase A, composed of water, chelating and wetting agents, kaolin, sodium stearate and Saccharomyces/Grape Lees Ferment, was weighed and heated up to 75 °C under stirring. Hydroxyethylcellulose was then slowly added to the phase A under stirring. Phase B, composed of the lipidic ingredients, was heated up to 75 °C and added to the phase A under homogenization (3500 rpm) until the temperature reached 40 °C. Vitamin E, preservative and parfum were added as the last step.

**Table 5:** Optimum growth conditions for the bacteria found in the samples (GDA-GDD).

Bacterial Genus	Temperature Range(°C)	pH Range	Salt Range (%)	Oxygen	Mannitol Fermentation	References
***Bacillus***	4–49	6–9	0–10	aerobic	+	[58]
***Lysinibacillus***	10–45	5.5–9.5	5–7	aerobic	−	[59]
***Oceanobacillus***	10–40	6.5–10	3–10	aerobic	+/−	[60]

**Table 6:** Different size measured at the DLS between yeast cells of native, sonicated and lyophilized lees samples.

SAMPLES	FREEZE-DRYING	SONICATION	SIZE	PDI
NWL_0_	−	−	2945 ± 26	0.084
SWL_0_	−	+	1476 ± 64	0.442
NWL_1_	+	−	2447 ± 28	0.676
SWL_1_	+	+	1723 ± 35	0.291

**Table 7:** Different concentration of element in the samples of wine lees before and after treatments of sonication and centrifugation.

	ELEMENT ANALYSIS		
	NWLs (ppm)	Supernatant of the NWLs (ppm)	Supernatant of the SWLs (ppm)
Li	0.06 ± 20.29	0.20 ± 6.41	47.26 ± 3.12
B	58.18 ± 1.11	168.94 ± 1.35	236.22 ± 1.90
Na	68.98 ± 2.91	315.89 ± 1.18	729.07 ± 4.23
Mg	577.88 ± 2.07	1735.23 ± 1.55	2125.98 ± 2.94
P	10,888.45 ± 1.56	9146.64 ± 1.27	7862.77 ± 2.56
S	3593.28 ± 3.11	4928.72 ± 1.78	5383.26 ± 5.85
K	13,514.31 ± 1.74	19,195.52 1.68±	65,338.26 ± 3.23
Ca	2687.06 ± 1.45	1130.35 ± 1.34	7927.05 ± 2.49
Cr	0.43 ± 2.15	1.10 ± 1.12	1.39 ± 3.41
Mn	10.72 ± 1.13	8.48 ± 1.40	20.63 ± 2.80
Fe	11.48 ± 1.92	8.28 ± 2.03	4.49 ± 6.68
Cu	227.34 ± 1.74	588.19 ± 0.91	262.41 ± 2.67
Zn	5.16 ± 4.94	14.46 ± 2.47	45.54 ± 2.25
Rb	12.21 ± 1.14	35.03 ± 1.72	62.21 ± 2.80
Sr	9.11 ± 1.81	11.93 ± 0.70	25.66 ± 1.62
Ba	2.16 ± 1.25	3.25 ± 1.12	10.53 ± 1.85
Hg	0.00 ± 6.49	0.02 ± 5.91	0.00 ± 14.57

**Table 8:** Results of the antioxidant assays.

	ABTS		DPPH		FOLIN	FRAP
	IC50 (mg/mL)	μmol TE/g	IC50 (mg/mL)	μmol TE/g	mg GAE/g	mg TEA/g
NWL	0.107 ± 0.003	204.525 ± 4.756	0.457 ± 0.009	870.692 ± 17.961	81.333 ± 9.905	55.048 ± 6.419
SWL	0.068 ± 0.001	182.489 ± 2.643	0.228 ± 0.020	607.195 ± 64.368	136.533± 8.942	82.133 ± 8.987
TROLOX	0.004 ± 0.000	7.991 ± 0.190	0.003± 0.000	120.577 ± 14.872		

**Table 9:** Developed products with relative properties.

Cosmetic Formulation	pH	Physicochemical Stability		Challenge Test	Mean Irritation Index	
		Accelerated Stability	Long Term Stability		15 min	24 h
Solid facial cleanser	5.85	Stable	Stable	Passed	0	0
Facial cleansing powder	5.84	Stable	Stable	Passed	0	0
Make-up remover	6.08	Stable	Stable	Passed	0	0
Melting mask	6.05	Stable	Stable	Passed	0	0
Purifying mask	5.80	Stable	Stable	Passed	0	0

## Data Availability

Data is unavailable due to privacy or ethical restrictions.
